# Facilitators and Barriers to Effective Smoking Cessation: Counselling Services for Inpatients from Nurse-Counsellors’ Perspectives — A Qualitative Study

**DOI:** 10.3390/ijerph110504782

**Published:** 2014-05-06

**Authors:** I-Chuan Li, Shoou-Yih D. Lee, Chiu-Yen Chen, Yu-Qian Jeng, Yu-Chi Chen

**Affiliations:** 1 Institute of Clinical and Community Health Nursing, School of Nursing, National Yang-Ming University, No. 155, Section 2, Li-Nong St. Beitou, Taipei 11221, Taiwan; E-Mail: icli@ym.edu.tw; 2 Department of Health Management and Policy, School of Public Health, University of Michigan, Ann Arbor, 1420 Washington Heights, Ann Arbor, MI 48109, USA; E-Mail: sylee@umich.edu; 3 Department of Nursing, School of Nursing, National Yang-Ming University, No. 155, Section 2, Li-Nong St. Beitou, Taipei 11221, Taiwan; E-Mail: chiuyenn@gmail.com; 4 Jhubei City Health Center, Public Health Bureau, HsinChu County Government, No. 89, Guangming 2nd St., Zhubei City, Hsinchu County 30251, Taiwan; E-Mail: accsohg@hotmail.com

**Keywords:** smoking cessation, counsellor, nurse, inpatient smoker, qualitative study, in-depth interviews

## Abstract

Tobacco use has reached epidemic levels around the World, resulting in a world-wide increase in tobacco-related deaths and disabilities. Hospitalization presents an opportunity for nurses to encourage inpatients to quit smoking. This qualitative descriptive study was aimed to explore nurse-counsellors’ perspectives of facilitators and barriers in the implementation of effective smoking cessation counselling services for inpatients. In-depth interviews were conducted with 16 nurses who were qualified smoking cessation counsellors and who were recruited from eleven health promotion hospitals that were smoke-free and located in the Greater Taipei City Area. Data were collected from May 2012 to October 2012, and then analysed using content analysis based on the grounded theory approach. From nurse-counsellors’ perspectives, an effective smoking cessation program should be patient-centred and provide a supportive environment. Another finding is that effective smoking cessation counselling involves encouraging patients to modify their lifestyles. Time constraints and inadequate resources are barriers that inhibit the effectiveness of smoking cessation counselling programs in acute-care hospitals. We suggest that hospitals should set up a smoking counselling follow-up program, including funds, facilities, and trained personnel to deliver counselling services by telephone, and build a network with community smoking cessation resources.

## 1. Introduction

Tobacco use has reached epidemic levels in many developing countries and steady use has continued in some developed nations [1,2,3]. Consequently, tobacco-related disabilities have increased worldwide, and the number of deaths caused by tobacco use (primarily cigarettes) has reached approximately 5 million each year around the World [4]. Taiwan has also been affected by the tobacco epidemic. In 2012, approximately 18,800 Taiwanese people died from smoking-related diseases, and the estimated population-attributable fraction of all-cause mortality resulting from smoking among Taiwanese citizens aged from 40 to 79 years was 27.8% for men and 6.7% for women [5].

There is a worldwide appeal to improve nurses’ involvement in tobacco control, particularly in cessation interventions [6]. Compared with other health professionals in acute-care hospitals, nurses constitute the largest workforce and are in more frequent contact with inpatients. Moreover, patient education and preventive healthcare are an integral component of nursing education [6,7]. Numerous studies have supported the role and efficacy of nurses in delivering smoking cessation counselling [8,9,10,11,12]. A clinical trial involving nurse case-managed tobacco cessation counselling for inpatients showed that patients receiving counselling intervention were more likely to abstain from using tobacco (28%) than those receiving only pharmacotherapy (16%) [11]. Another study indicated that intensive nursing counselling with telephone follow-ups was more effective than brief counselling and providing educational materials alone [12]. The environment setting where cessation interviews are conducted is also relevant. Several previous studies have reported a high level of motivation among patients to quit smoking during hospitalization [13,14]. The restricted or no-smoking policies in hospitals interrupt patients’ smoking habits, creating a ‘teachable moment’ for inpatients who smoke [8,9,13]. Rice and Stead asserted that hospitalization creates an opportunity for nurses to provide intensive smoking cessation counselling for patients diagnosed with smoking-related illnesses.

### Background

The Taiwanese Health Promotion Administration (THPA) reported that there were approximately 4.8 million smokers in Taiwan, and that approximately 47% of them had considered quitting at least once. The success rate of quitting smoking, however, is less than 5% [15]. Quitting tobacco use benefits the majority of smokers and their loved ones; it saves lives and reduces personal and health care costs [16,17]. Since 2002, the Taiwanese government has initiated several outpatient clinic programs in hospitals to encourage smokers to quit. These programs integrate smoking cessation counselling into routine outpatient visits. All of the programs offer financial incentives to doctors who provide outpatients with nicotine replacement therapy (NRT) and brief smoking cessation counselling during routine visits. Although the financial incentives have resulted in an increase in the number of patients who have received smoking cessation counselling from physicians, the smoking cessation rate remains unsatisfied [18,19]. The percentage of smokers that attempted to quit declined slightly in 2007 after the funding was reduced in 2006 [18].

An analysis of the 2004–2007 Taiwan Adult Tobacco Surveys showed that 71% of doctors in Taiwan had failed to offer smoking cessation counselling to patients, a rate that was higher than those reported in the United Kingdom (59%) and the United States (39%) [18]. Because Taiwanese physicians typically serve more than 50 outpatients in a single morning or afternoon work shift, time limitations have been a major barrier to counselling patients on smoking cessation [18,19]. Moreover, physicians typically provide only NRT; they seldom address patients’ psychological dependence on nicotine, which contradicts previous research findings indicating that the success rate of quitting smoking is high among smokers who receive intensive advice or counselling [20].

The THPA implemented the New Smoking Cessation Policy on the 1st of March 2012 to expand smoking cessation counselling services to both outpatient and inpatient settings. The new policy is based on the following three evidence-based premises: (i) non-physician providers (specifically nurses) have an essential role in providing smoking cessation counselling; (ii) increasing the intensiveness of counselling services may improve the success rate of smoking cessation; and (iii) inpatient settings may facilitate intensive smoking cessation counselling. Hospitals that participate in the inpatient smoking cessation program send qualified employees, most of them nurses, to receive training to become smoking cessation counsellors. As a result of the expansion of smoking cessation counselling to inpatient settings and inclusion of nurses in the provision of cessation counselling services, the new policy has broadened the scope of effort to identify smoking patients when they are most receptive to tobacco cessation intervention as well as increased the range of patient-provider interface to allow patients be served by trained nurse-counsellors.

Previous research in the education sector showed that the perceptions of school nurses had a significant influence on school administrators and parents in the promotion of student health education [21]. However, few studies have elucidated the perceptions of nurse-counsellors regarding smoking cessation interventions. There is also a lack of information about factors that facilitate and inhibit hospitals nurses’ provision of smoking cessation counselling services. Although being in direct contact with patients’ places nurses in an advantageous and convenient position to counsel patients on quitting tobacco products, short periods of hospitalization, increased severity of patients in acute-care setting and constraints on hospital budget may limit the effectiveness of nurse-led smoking cessation counselling programs [6,16,17]. Thus, it is important to understand from nurse-counsellors’ perspective the operation and challenges of the new smoking cessation counselling program in order to improve program implementation and inform policy change.

In this study, we employed a qualitative study design and interviewed nurse-counsellors to elucidate their perspectives on smoking cessation counselling, as well as their perceptions on the facilitators and barriers of smoking cessation programs. Specifically, the following research questions were addressed:

(i) From the nurse-counsellors’ viewpoint, what were the factors that facilitate the provision of smoking cessation counselling services to inpatients?
(ii) From the nurse-counsellors’ viewpoint, what were barriers in the provision of smoking cessation counselling services to inpatients?

The findings of this study may assist health care providers implementing smoking cessation programs and provide a reference for the development of effective training programs for smoking cessation counsellors.

## 2. Methods

### 2.1. Study Aim

The aim of the study was to explore the perspectives of nurse-counsellors regarding the facilitators and barriers to implementing effective smoking cessation counselling services for inpatients.

### 2.2. Study Design

This qualitative descriptive study employed a grounded theory approach. We conducted in-depth interviews to explore the perspectives of nurse-counsellors regarding the environment of hospitals in which inpatient smoking cessation programs were implemented as well as their experiences in delivering inpatient smoking cessation counselling services. Previous studies have asserted that qualitative research methodologies are effective for examining novel research topics because they can assist in soliciting information from service users or providers to develop and improve health interventions through in-depth probing with questions that are meaningful and context-specific to key informants [22,23]. It could also contribute to improving novel treatment programs through recognition of the experiences and opinions of relevant health professionals and patients [23]. Because nurse-counsellors work closely with smokers, they have an intimate understanding of the challenges that affect patients, as well as the insight regarding the necessary organizational conditions that facilitate successful counselling programs. Hence, their perceptions are critical for evaluating and improving the novel inpatient smoking cessation programs.

### 2.3. Participants and the Recruitment Procedure

The participants in this study were nurses who had (i) provided cessation counselling services to inpatients, (ii) received at least one of three-levels of training workshop on smoking cessation, and (iii) received counselling certification from the THPA. Nurses who meet these three criteria are called nurse-counsellors and the smoking cessation counselling services they provide are eligible for reimbursement by THPA.

Beginning in May 2012, we approached directors of departments that were responsible for the delivery of smoking cessation services at 25 smoke-free health promotion hospitals in the Greater Taipei City Area (including Taipei City and new Taipei City) in northern Taiwan and asked them to recommend nurse-counsellors. Seventeen directors agreed to our request and indicated that their hospital had one or two nurse counsellors that were eligible as study participants in accordance to our selection criteria. We contacted those nurse-counsellors to invite their participation, and 16 agreed to be interviewed. We arranged an interview at a time and a place convenient to those who agreed. By October 2012, we had interviewed all 16 nurse-counsellors at 11 health promotion hospitals and determined that the information we obtained had reached saturation. These 16 nurse-counsellors from 11 health promotion hospitals constituted the study sample.

### 2.4. Data Collection and Analysis

For the in-depth interviews, we developed semi-structured open-ended questions based on field observations and a review of extant literature. The questions were designed to investigate (i) facilitating factors that the nurses considered effective in helping patients to quit smoking, and (ii) barriers that nurse-counsellors experienced while delivering smoking cessation services. The interviews were conducted by two authors (CYC was a PhD candidate and YQJ had a master degree) with relevant experience in qualitative research methods and interviewing. Prior to collecting the data, one of the investigators (YCC) led a pilot study with the two interviewers (CYC & YQJ) to test the interview guide and questions. The results of the pilot test were discussed among the members of the research team and subsequently applied to improve the interview guide (Table 1).

**Table 1:** The interview questions and guides.

Interview questions
Tell me about your experience in performing smoking counseling services to in-patients smokers.
What facilitating factors are you consider effective in helping inpatients to quit smoking?
Any barrier you experienced while you delivering smoking cessation counseling services in hospitals?

Within three days of completing each interview, the recorded interviews were transcribed verbatim by one of the interviewers, and the transcripts were reviewed by the other one to eliminate errors. Information that was considered ambiguous was clarified with the participants. 

We conducted a content analysis using the principles of the grounded theory approach. Grounded theory is an inductive research approach; it involves the discovery of theory from ground up, through the analysis of in-depth interview data [24]. The analysis was performed using Atlas.ti 6.2. First, we coded all of the highlighted passages that were considered relevant to the research questions. Second, the codes were compared and sorted into meaningful categories based on the key concepts of this study (*i.e.*, strategy, effectiveness, and barriers). Third, the categories were divided into subthemes and themes, which were independently compared and reconciled by three of the authors. Finally, the authors discussed the main themes that emerged from their analyses, and compared the categories to resolve discrepancies. The review and comparison process was iterative, involving the review of specific transcripts in their entirety.

### 2.5. Rigour

Throughout the data analysis, the authors worked collaboratively and iteratively to develop and revise the codes and categories. Following the process of reflection and discussion, a consensus was reached on the appropriate and meaningful approach to sorting and grouping the codes, as well as formulating the categories into subthemes and main themes.

There are four issues of trustworthiness that demand attention in qualitative research: credibility, transferability, dependability, and conformability [25]. To increase credibility, interviews were independently coded. Other strategies include member checking (clarifying with participants during the interviews) and peer checking (discussing data until a consensus was reached). Furthermore, all of the authors had experience in tobacco cessation and qualitative research, which increased the credibility of the study. Transferability was met through careful selection of participants using explicit inclusion and exclusion criteria. Dependability was ensured by the investigator’s completion of verbatim transcriptions from audiotapes in a timely manner. Conformability was achieved by maintaining an audit trail and research reflection log.

### 2.6. Ethical Considerations

The research protocol was approved by the Institutional Review Board at National Yang-Ming University in Taiwan. The IRB approval number is 2012-07-036 BCY. Prior to conducting the interviews, we informed the participants regarding the research purpose and procedures, as well as their rights. All of the participants signed an informed consent form, with an understanding that their participation was entirely voluntarily. We also had the participants’ permission to audio-record the interview. To assure anonymity, we used a unique numeric identifier to replace the name of each participant on the documents of study and the citations of results.

## 3. Results

Participant characteristics are reported in Table 2. The mean age of the 16 interviewed nurse-counsellors was 36.5 years (SD = 9.8) and all of them were women. Half of them were university graduates, and all reported that they had successfully completed formal training on smoking cessation counselling. None of them had a history of smoking. The mean number of years working as a registered nurse was 12.7 (SD = 11.0), and the mean number of years being involved in smoking cessation counselling was 3.0 (SD = 2.3). They all claimed to provide smoking cessation counselling services in accordance to the guidelines designed by the TBHP in Taiwan. Our content analysis revealed the following themes and subthemes.

### 3.1. Theme 1: Facilitators that Enhance the Success of Smoking Cessation

Hospitalization is a crucial moment or occasion that triggers patients’ motivation to quit smoking. The following facilitators emerged from the interviews with nurse-counsellors: (i) building work teams to create a supportive smoking cessation environment; (ii) shifting the focus to the modification of smokers’ lifestyles; (iii) selecting an appropriate time to initiate counselling; (iv) providing patient-centred counselling; and (v) referring patients to other resources when necessary.

**Table 2:** The characteristics and demographics of the participants.

Items	n (%)/Mean (±SD)
**Age**	36 (±9.8)
**Gender**	
Male	0 (0.0%)
Female	16 (100%)
**Education**	
College	7 (43.8%)
University	8 (50.0%)
Graduate	1 (6.2%)
**Registered nurse work experience (year)**	12.7 (±11.0)
**Smoking cessation counselor position**	
Full time	11 (68.8%)
Part time	5 (31.2%)
**Experience as a smoking cessation counselor (years)**	3.0 (±2.3)
**Smoking experience**	
Yes	0 (0%)
No	16 (100%)

#### 3.1.1. Collaboration with Other Health Professionals to Develop a Supportive Cessation-Oriented Environment

When health professionals work cooperatively in emphasizing the benefits of quitting smoking to patients during patient interactions, they are more effective at promoting smoking cessation counselling and motivating patients to quit smoking. This assessment, exemplified by the following responses, was shared among all of the interviewed nurse-counsellors:

‘*If all hospital staff—including professional health workers, paraprofessionals, and volunteers—who meet with the patients emphasize that smoking has caused their illness and [if the staff] inform them that quitting smoking would be beneficial for treating their diseases, the patients would have the strongest motivation to quit smoking.*’ *(Counsellor B)*

‘*When a patient comes to the hospital reporting chest pain, a doctor could ask them if they [smoke]. If they confirm that they [smoke], the doctor should [inform] the patient of the correlation between chest pain and smoking…afterward, the pathology staff may ask these patients whether they [smoke], and then advise them to quit during the process of physical examination.*’ *(Counsellor A)*

Moreover, the majority of the interviewed counsellors emphasized the importance of employing a team approach to creating a cessation-oriented environment in order to effectively help patients to quit smoking:

‘*For patients to quit smoking, they require a whole team of health professionals to support and assist them.*’ *(Counsellor N)*

‘*Collaboration is the best strategy for all hospital health professionals, including physicians, nurses, medical equipment operators, pharmacists, and even health volunteers and priests because a positive and supportive smoking cessation environment helps inpatients quit [smoking].*’ *(Counsellor D)*

#### 3.1.2. Timing for Initiating Smoking Cessation after Hospitalization

To improve the success rate of patients quitting smoking, the nurse-counsellors indicated that the timing for initiating counselling services was a crucial aspect of effective smoking cessation strategies:

‘*[It is crucial] to contact smokers as quickly as possible when they are admitted [to] the hospital…frontline nurses and physicians should immediately provide advice if the patients report that they are smokers during the admission assessment of [newly] admitted patients…, which gives them the best opportunity to provide counselling services for smokers.*’ *(Counsellor A)*

‘*If primary nurses inquire about the patients’ smoking history when they are admitted, then physicians should also tell them that [whether their condition] is related to smoking, offer advice on quitting, and refer [them] to [smoking] cessation counsellors. That would improve the patients’ intention to use cessation service.*’ *(Counsellor J)*

‘*If we have good timing, then patients are more likely to be willing to quit.*’ *(Counsellor D)*

‘*Usually, we contact [newly] admitted patients within 24 hours after their primary nurses have marked them as smokers... if we do not [attend to smokers] punctually, they will be discharged soon. Then, we [would miss a] good chance [to intervene].*’ *(Counsellor B)*

#### 3.1.3. Modifying a Smoker’s Lifestyle is Essential

To assist smokers in quitting, it is critical that healthcare providers promote a healthful lifestyle that includes regular exercise, a healthy balanced diet, and stress management. Several of the nurse-counsellors reported the following observations:

‘*Smoking is not only a physiological dependency but is also a psychological dependency. [Moreover], the psychological dependency is the primary reason that smokers continue [to smoke]; even when they have [been provided] the necessary advice, health information, and motivation to [modify their behaviour], it is easy for them to relapse if they do not change their lifestyle... Smokers must adjust [their] behaviour and lifestyle to cope with the stress [associated with quitting]. Moreover, they require a therapist for psychological treatment.*’ *(Counsellor F)*

‘*Healthcare providers should not only emphasize how crucial quitting smoking is for promoting patient’s health, but they should also shift their emphasis to other health-promoting behaviours such as exercising, maintaining a healthy diet, and managing stress.*’ *(Counsellor D)*

#### 3.1.4. Application of Patient-Centred Techniques, Useful Information, and Teaching Resources

Although the time constraint appeared to be universal, the interviewed nurse-counsellors advocated that the intervention approach—specifically, providing patients with information and education on quitting smoking—must be individualized and patient-centred to address each patient’s specific needs.

‘*It is useless if the counsellors educate smokers on quitting based only on their professional knowledge and do not assess smokers’ specific needs and difficulties.*’ *(Counsellor P)*

‘*[Quitting smoking], I think, is an individual experience. The situation varies among patients…There are different stages for smoking cessation. Healthcare providers need to provide different patients with different things; you need to help them identify better ways [to quit] by providing patients with the appropriate information for their stage [of quitting].*’ *(Counsellor A)*

‘*Providing patients with various educational materials that [are] relevant to the patient’s stage of quitting…Some patients are still hesitant; they need to [be more determined]…Those who have decided to quit should go directly to the process [commence counselling immediately]; that is, the patient needs information on how to quit smoking and the type of problems they [will] face.*’ *(Counsellor D)*

#### 3.1.5. Referring Patients to Other Counselling Resources

Consistent with the patient-centred approach, the interviewed nurse-counsellors recognized that patients may have special needs that could be better addressed elsewhere or with alternative resources. Thus, referrals to other appropriate smoking cessation resources are necessary when a counsellor feels unqualified to address a specific question or the personnel, knowledge, or resources in the hospital are limited:

‘*When I encountered something I did not know, I did not answer the patients; rather, I provided a hotline for quitting smoking.*’*(Counsellor M)*

‘*When there are some problems we do not know the answers to, we try to find an answer. I often read a research paper or ask professionals.*’*(Counsellor O)*

‘*Professional counsellors must learn constantly and accumulate experience to become more confident and capable of identifying solutions and resources [that can] assist their patients. If they are [neither confident nor capable], then they have to make a referral.*’ *(Counsellor K)*

### 3.2. Theme 2: Barriers to Providing Counselling Services

The nurse-counsellors were confronted with substantial organizational-level barriers, including a lack of full support from the hospital administration, a lack of commitment from other professionals, as well as a shortage of time and personnel.

#### 3.2.1. Lack of Full Support from Hospital Administration

Although it is recognized that the promotion of a supportive smoking cessation environment in hospitals can improve the successful delivery of smoking cessation counselling services, the nurse-counsellors indicated that there was a lack of complete support from hospital administration:

‘*The cost of providing this type of counselling service for smoking cessation, which is allocated by the administrators, is very low.*’ *(Counsellor H)*

‘*If an administrator always mentions the smoking cessation program during the hospital meeting and [informs the] physicians to advise patients to quit, then the program can go on providing service to patients well and the program will succeed.*’ *(Counsellor O)*

‘*Just a little more support [for the smoking cessation program] from the managers would be sufficient.*’ *(Counsellor E)*

#### 3.2.2. Insufficient Time and Personnel Support in Hospitals

The nurse-counsellors reported that their time and effort were stretched thin.

‘*Because we are short of staff, the nurses are already [overloaded] with tasks. It is impossible to provide patients with all of their health education needs. [The trained nurse-counsellors] really do not have time to persuade patients not to smoke.*’ *(Counsellor H)*

‘*Most of the counsellors in hospitals have several duties. We not only need to follow various cases and [manage] new cases, but we must also provide regular care to other inpatients... Usually, we feel unwilling to do this because we are not full-time counsellors.*’ *(Counsellor O)*

‘*Because we lack a sufficient number of nursing staff, we often work for many [consecutive] days. We sometimes need to work night shifts, and we occasionally work for more than 12 hours... It is really difficult to allocate time to do this. In fact, it is a difficult job.*’ *(Counsellor L)*

‘*If [counsellors were employed] full-time, [then they would] do a much better job. We can follow the cases after we discharge the patients from the hospital to provide health education and guidance on how to prevent them from relapsing…*’ *(Counsellor N)*

#### 3.2.3. Lack of Commitment from Other Health Professionals

Consistent with the aforementioned subthemes, other hospital health professionals, such as doctors, social workers, nutritionists, and pharmacists, were unaware of how crucial and effective their advice was for smokers during hospitalization. Consequently, the following nurse-counsellors’ reports indicated that there was a general lack of clear commitment from these health professionals:

‘*The advice provided by doctors is very useful for helping smokers to quit. However, not every doctor has the incentive to advise smokers… [Similarly], primary care nurses typically do not think they are responsible for advising patients on smoking cessation and do not consider advising patients to be a part of their duty.*’ *(Counsellor F)*

‘*Most physicians are not interested in providing smoking cessation service—even brief advise… they won’t receive any [funding] from National Health Insurance [for offering these services], so they lack the financial incentive.*’ *(Counsellor C)*

## 4. Discussion

This is the first study in Taiwan to understand from nurse-counsellors’ perspective facilitators and barriers for smoking cessation counselling services in inpatient settings. A total of sixteen trained nurse-counsellors from 11 acute hospitals participated in this study. They proposed several facilitators to effectively promote and implement smoking cessation counselling programs: collaborate with other health professionals in hospitals to create a supportive cessation-oriented environment, visit smoking inpatients and provide them counselling services as quickly as they are admitted, modify smokers’ behaviours to promote a healthy lifestyle, apply patient-centred techniques in smoking cessation counselling services, and refer hospitalized smokers to other counselling resources when they are discharged from hospitals. From nurse-counsellors’ perspectives, barriers to implement smoking cessation services include the lack of full support from hospital administration, insufficient time and personnel support, and lack of commitment from other health professionals in hospitals.

Although hospitalization provides a window of opportunity for smoking cessation [16,17], delivery of cessation counselling services is challenging. In addition to the aforementioned barriers, hospitalized patients have increased acuity that needs intensive treatment and smoking cessation is secondary to their medical priorities. This perhaps explains why nurse-counsellors pointed out consistently that timing and a comprehensive approach involving the entire hospital to promote smoking cessation are critical for the success of the smoking cessation counselling services program. Ideally, when patients are admitted to hospital, nurses should determine whether they smoke, and immediately initiate smoking cessation counselling services for those who do. Previous studies similarly indicated that routine medical charting during the admission process should include smoking assessment, thereby assisting healthcare providers in identifying smokers and initiating smoking cessation services [26]. New efforts that capitalize on the use of electronic medical records (EMRs) by adding a smoking module to the EMR system to make the inquiry of patients’ smoking status a routine procedure may increase quitting among the patient population [27].

From nurse-counsellors’ perspective, a comprehensive counselling program must include lifestyle modifications (e.g., healthy diet, regular exercise, and stress management), rather than providing only health education and pharmacotherapy for physiological dependence. This idea is consistent with other studies. May *et al.* [28], Kotz *et al.* [29], and Chang and Wang [30] have contended that smoking cessation is a behaviour modification process, and that it requires personal motivation to be succesful. Current approaches to smoking cessation recognize that cigarette smoking is not a simple habit, but a complex physiologic addiction that requires a multi-faceted intervention scheme from a variety of sources in order to encourage lifestyle change to overcome nicotine addiction and prevent relapse [31,32]. Hospital nurses’ practice of health education, through individual nurse-patient interactions, can make an important contribution to health promotion [7]. However, short hospital stays and changing patterns of patient care make it difficult to combine nursing care with patient-oriented and time-intensive behavioural interventions [6,17]. This is one of the barriers to effective smoking cessation counselling services reported by nurse-counsellors in this study.

The interviewed nurse counsellors all reported feeling frustrated as a result of several barriers, all of which were directly or indirectly related to the lack of support and resources for delivering smoking cessation services in hospitals. Consequently, the nurse-counsellors reported having insufficient time and inadequate competence to provide these services, which supported the findings reported by Sarna *et al.* [6]. In Sarna *et al.*’s study, the barriers identified by nurses involved in providing smoking cessation services included a lack of competence, referral options, and resources, as well as time constraints. Two other studies showed that nurses felt competent and were effective at assisting smokers to quit when they were able to commit full time to providing smoking cessation services [8,33]. A meta-analysis also indicated that brief smoking cessation counselling provided by nurses who combined smoking cessation work with other duties were less effective than long-period interventions delivered by nurses with a full-time role in health promotion [8]. Having sufficient staff time to provide smoking cessation services also appears to be a key factor of successful cessation programs.

The interviewed nurse-counsellors reported concerns regarding the commitment of other healthcare providers to providing inpatient smoking cessation services. Physicians, for example, did not consider offering advice to patients on quitting smoking as a part of their duty, partially because they were doubtful of the effect of their advice and their patients’ receptivity. Another reason could be that the assessment of their patients’ smoking status had not been incorporated into routine care processes [26]. A study in Hong Kong found that smoking cessation practice was influenced by healthcare providers’ attitudes and expectations of their roles and their self-perceived competence. Educating healthcare providers about the importance and effectiveness of smoking cessation programs might encourage them to become involved in delivering these services [34,35]. Multidisciplinary collaboration among healthcare providers may foster a sense of collective competence and increase providers’ commitment to smoking cessation services [34].

Based on comments of the interviewed nurse-counsellors, future administrative support and resources must be invested to: (i) educate healthcare providers, (ii) implement a hospital-wide smoking cessation policy that incorporates the assessment of smokers into routine care practices, (iii) develop health education materials for patients, (iv) allow sufficient time for healthcare professionals to provide smoking cessation counselling to their patients, and (v) establish a referral system for patients to receive smoking cessation services. Furthermore, follow-up counselling services are needed to reduce relapse of smoking patients after their discharge from the hospital. A Cochrane review on studies of hospitalized smokers suggests that interventions need to last at least one month post-discharge to have a statistically detectable effect [17]. An alternative approach to continuing the counselling is through telephone, which is convenient and can be delivered proactively. Similar to inpatient counselling services, the design of telephone counselling would also need to be tailored to the specific needs of individual smokers.

Several features of this study design need to be discussed. First, the study sample is small. Considering the exploratory nature of the study, the small sample size may be justifiable. Second, the interviewed nurse-counsellors were from different hospitals, which may influence nurse-counsellors’ perceptions of smoking cessation programs. It is important to note, however, all the programs served similar patient groups and were located in hospitals that shared the same mission of health promotion. To the extent that organizational attributes of hospitals affected the implementation and effectiveness of smoking cessation counselling services, a high degree of consistency in the perceptions and recommendations of nurse-counsellors interviewed suggests that findings of the study are robust and have good generality to other hospitals in Taiwan. Finally, the nurse-counsellors interviewed in the study were referred by their supervisor in the hospital. They were likely to be highly motivated and actively involved in the inpatient smoking cessation counselling program. Our findings may be limited by not including nurse-counsellors who were not fully committed to the counseling program and who may have provided useful information that highlight additional barriers that prevent their active involvement in the smoking cessation counselling program.

## 5. Conclusions

Hospitalization provides a unique opportunity for nurses to deliver smoking cessation counselling services. There is a broad consensus among nurses-counsellors who provide smoking cessation counselling that more emphasis should be placed on lifestyle changes than on pharmacotherapy. Nurse-counsellors believe that a supportive environment, teamwork, and provision of patient-centred care are key elements of successful smoking cessation programs for inpatients. Barriers to smoking cessation services exist primarily at the organizational level; they include the lack of full support from hospital administration level, insufficient time and personnel support, and lack of commitment from other health professionals in hospitals.

The study has several practice implications. First, nurse-counsellors must assess the needs of smokers throughout the cessation process by using more advanced technology like electronic medical records. Second, to increase the involvement of other healthcare professionals, they need to be informed on the smoking cessation program and its effectiveness. Third, nurse-counsellor training programs should include modules on health education and behavioural modification to improve their competence in helping smokers to quit. Finally, increasing the reimbursement for smoking cessation services might encourage hospital administrators to allocate additional resources to smoking cessation programs. For hospital administrators, they may consider the provision of follow-up counselling services to discharged patients. Establishment of a network with community smoking cessation resources is another strategy to continue the smoking cessation services post-discharge.

A report issued by the Health Behaviour Export Panel of the American Academy of Nursing (AAN) advocated efforts to increase nurses’ visibility and influence in tobacco control services [36]. The inpatient smoking cessation counselling program, in a way, is designed to heed the call of the ANA expert panel. The program is nurse-led and nurse-delivered. It demonstrates that nurses, indeed, can be trained to take a lead in providing smoking cessation counselling services and that they play a unique role in inpatient smoking cessation because of their frequent contact with patients and their “professional instinct” to pay attention to individual needs of patients. Almost all the facilitators and barriers revealed in the interviews with nurse counsellors’ organizational or system-wide factors. We suspect that those factors are prevalent in hospitals and health care organizations in other countries.

Another important finding of the study is recognition among nurse counsellors that inpatient smoking cessation services are but the beginning of a patient’s long journey of ridding a hardwired habit. The occasion of hospitalization opens a window of opportunity. The opening is slim, however, and it is likely to become smaller as cost-cutting efforts in many countries are reducing the length of hospital stays. Continuing interventions that extend beyond the hospital stay are needed and they would require the investment of hospitals in follow-up services and in building collaboration with outpatient clinical settings and community-based social service agencies and advocacy groups.

Based on the study findings, we urge that hospitals, community organizations, nursing organizations, and governmental funding agencies work together to ensure that nurse-counsellors be involved in tobacco control network. Smoking cessation training program should provide nurse-counsellors the skills to mobilize community resources. We also recommend future research to examine the various designs of inpatient smoking cessation counselling. An example of such research is the application of electric medical records in smoking cessation programs, which may enhance the identification and recruitment of smoking inpatients and improve the coordination of nurse-counsellors with other health professionals.
